# *In Vivo* Modelling of *ATP1A3* G316S-Induced Ataxia in *C*. *elegans* Using CRISPR/Cas9-Mediated Homologous Recombination Reveals Dominant Loss of Function Defects

**DOI:** 10.1371/journal.pone.0167963

**Published:** 2016-12-09

**Authors:** Altar Sorkaç, Ivan C. Alcantara, Anne C. Hart

**Affiliations:** Department of Neuroscience, Brown University, Providence, RI, United States of America; INSERM U869, FRANCE

## Abstract

The NIH Undiagnosed Diseases Program admitted a male patient with unclassifiable late-onset ataxia-like symptoms. Exome sequencing revealed a heterozygous *de novo* mutation converting glycine 316 to serine in *ATP1A3*, which might cause disease. *ATP1A3* encodes the Na^+^/K^+^ ATPase pump α3-subunit. Using CRISPR/Cas9-mediated homologous recombination for genome editing, we modelled this putative disease-causing allele in *Caenorhabditis elegans*, recreating the patient amino acid change in *eat-6*, the orthologue of *ATP1A3*. The impact of the mutation on *eat-6* function at the neuromuscular junction was examined using two behavioural assays: rate of pharyngeal pumping and sensitivity to aldicarb, a drug that causes paralysis over time via the inhibition of acetylcholinesterase. The patient allele decreased pumping rates and caused hypersensitivity to aldicarb. Animals heterozygous for the allele exhibited similar defects, whereas loss of function mutations in *eat-6* were recessive. These results indicate that the mutation is dominant and impairs the neuromuscular function. Thus, we conclude that the *de novo* G316S mutation in *ATP1A3* likely causes or contributes to patient symptoms. More broadly, we conclude that, for conserved genes, it is possible to rapidly and easily model human diseases in *C*. *elegans* using CRIPSR/Cas9 genome editing.

## Introduction

The Undiagnosed Diseases Program of the National Institutes of Health (NIH) aims to identify the cause of patient symptoms that are not explained by a known syndrome (also called rare diseases)[1]. Under this program, exome sequencing was undertaken for a patient suffering from numerous neurological symptoms including ataxia. This revealed a *de novo* G316S mutation in the fourth transmembrane domain of the ATP1A3 protein. The mutation is predicted to impact protein function and potentially cause patient symptoms. Recently, the G316S mutation was shown to reduce pump activity when *ATP1A3* was overexpressed *in vitro*[2].

*ATP1A3* encodes an alpha 3 subunit (ATP1α_3_) of sodium/potassium ATPase 1 (Na^+^/K^+^ ATPase 1). Na^+^/K^+^ ATPases use energy from hydrolysis of ATP to synchronously pump sodium out and potassium into the cell, creating a membrane potential based on different concentrations of these cations inside and outside the cell. Establishment of membrane potentials and ionic concentration gradients is crucial for excitable cells including glia, neurons and muscles. Although Na^+^/K^+^ ATPases are required in virtually every cell, the alpha 3 subunit is predominantly expressed in neurons[3].

Mutations in ATP1A3 have been associated with a number of neurological syndromes, including rapid-onset dystonia parkinsonism (RDP) and alternating hemiplegia of childhood (AHC)[4]. ATP1A3 mutations that cause RDP are dominant and decrease ATP1A3 levels in HEK293 cells[5]. Mutations causing AHC do not seem to affect protein levels; however ATPase function is decreased[6]. Patient symptoms, however, did not perfectly fit either of these diagnoses.

To determine impact of G316S mutation on ATP1A3 *in vivo*, we studied the effect of the mutation on the protein function in *C*. *elegans*. For this analysis, we introduced the analogous G-to-S mutation in the *C*. *elegans* orthologue, EAT-6. Previous work demonstrates that *C*. *elegans* can be used to create reliable *in vivo* models for neurological diseases[7]; however most of these models are based on overexpression of mutant human proteins[8–12].

EAT-6 is orthologous to ATP1A3 and the affected glycine residue is in a well-conserved region of EAT-6. *eat-6* was originally identified as a pharyngeal pumping defective mutant in 1993[13] and subsequently found to encode a Na^+^/K^+^ ATPase subunit[14]. *eat-6* partial loss of function mutations lead to mislocalisation of nicotinic acetylcholine receptors and to hypersensitivity to inhibitors of cholinesterase[15, 16].

To recreate G316S in *C*. *elegans eat-6*, we took advantage of the CRISPR/Cas9-mediated homologous recombination (HR) technology. The system was adapted to *C*. *elegans* for genome editing through creation of insertions/deletions (indels)[17]. Since then, numerous *C*. *elegans* groups have reported modifications to the system, including CRISPR-Cas9 mediated homologous recombination[18]. Circular plasmids[18], linear PCR products or single-stranded oligonucleotides (ss oligos)[19] have been successfully used in *C*. *elegans* as templates for homologous recombination. CRISPR/Cas9-mediated HR in *C*. *elegans* allows inexpensive, fast and reliable engineering of the genome, providing the means to study the effect of different mutations on gene function. To analyse the effect of the patient mutation on the protein function, we created the homologous G304S mutation in EAT-6 and examined if the signalling at the neuromuscular junction was altered.

## Results

**Fig 1:**
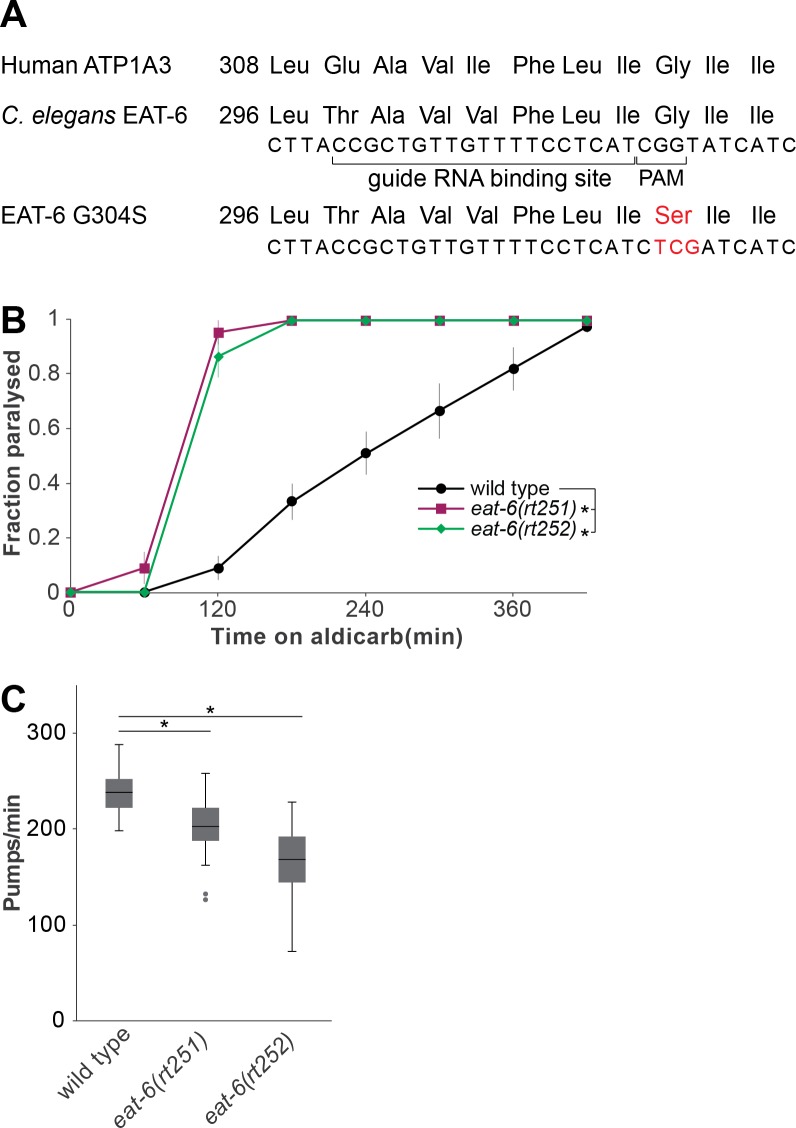
EAT-6 G304S mutation is analogous to ATP1A3 G316S and perturbs neuromuscular junction function. (A) EAT-6 is homologous to ATP1A3 and glycine 304 (G304) is in a well-conserved region. The PAM site and the sgRNA binding site used for EAT-6 G304S (red) are marked with brackets. (B) For the two alleles of *eat-6* isolated independently (*eat-6(rt251)* and *eat-6(rt252)*), G304S caused hypersensitivity to aldicarb (3 trials ± SEM, *: log-rank p-value<0.05). (c) *eat-6* G304S animals exhibited decreased pumping rates (median, flanking quartiles in Tukey format; 3 trials; *: Mann-Whitney U-test (two-tailed) p-value<0.05; n.s.: not significant, p-value>0.05).

To model ATP1A3 G316S in *C*. *elegans*, we aligned the amino acid sequences of the human ATP1A3 and EAT-6 (S1 Fig). This revealed 83% similarity and 72% identity across the full length proteins (NP_689509.1 and B0365.3, EMBOSS-Needle) and the glycine residue at position 304 of EAT-6 was found to correspond to ATP1A3 G316 (Fig 1A). Hence, we sought to change the *C*. *elegans* G304 to serine.

The protospacer adjacent motif (PAM) is required for *S*. *pyogenes* Cas9 binding and contains a GG sequence[20]. Since glycine codons contain GGs, the G304 codon was used as the PAM site. Furthermore, by converting the residue into serine and removing the PAM site, we protected the edited genomic sequence from further Cas9 cleavage. To create the EAT-6 G304S mutation we used a single stranded oligonucleotide template containing the recombinant sequence (Fig 1A).

Guide RNAs targeting a genome sequence with perfect complementarity can result in off-target mutations in similar sequences[21]. To control for possible background mutations, we isolated the EAT-6 G304S mutation twice, creating two independent alleles that were subsequently homozygozed and designated as *eat-6(rt251[G304S])* and *eat-6(rt252[G304S])*.

The partial loss of function reference *eat-6* allele, *ad467*, has been shown to reduce Na^+^/K^+^ ATPase activity [22]. *ad467* carries a leucine to phenylalanine mutation of the residue 359 in the intracellular loop between the 4^th^ and 5^th^ transmembrane domains [15]. L359 in *C*. *elegans* EAT-6 corresponds to L371 in the human protein, placed adjacent to the CSDK motif (S1 Fig). This allele causes hypersensitivity to aldicarb, an inhibitor of acetylcholinesterase that leads to acetylcholine accumulation at the neuromuscular junction and paralysis of *C*. *elegans* over time[15]. Hypersensitivity to aldicarb is indicative of defects in neuromuscular junction function. To determine whether the G304S mutation reduces protein function, we assessed paralysis rates on 1mM aldicarb. Both *eat-6(rt251)* and *eat-6(rt252)* were paralysed faster on aldicarb, compared to wild type controls (Fig 1B; p-value for *rt251* v. wild type = 1.344E-17, p-value for *rt252* v. wild type = 8.042E-16), suggesting that this amino acid change decreased Na^+^/K^+^ ATPase function.

To confirm that EAT-6 G304S decreases function, we examined impact on a different neuromuscular system. In *ad libitum* food conditions, *C*. *elegans* constantly ingest bacteria via rhythmic contraction of pharyngeal muscles. Loss of EAT-6 function reduces pharyngeal pumping rates[13]. Hence, if EAT-6 G304S decreases function, fewer pumps per minute would be observed. Consistent with this hypothesis, both G304S alleles resulted in reduced pumping rates compared to control animals (Fig 1C; p-value for *rt251* v. wild type = 4.913E-6, p-value for *rt252* v. wild type = 6.140E-13). This is consistent with aldicarb results and confirms that the G304S mutation likely causes a decrease in protein function.

**Fig 2:**
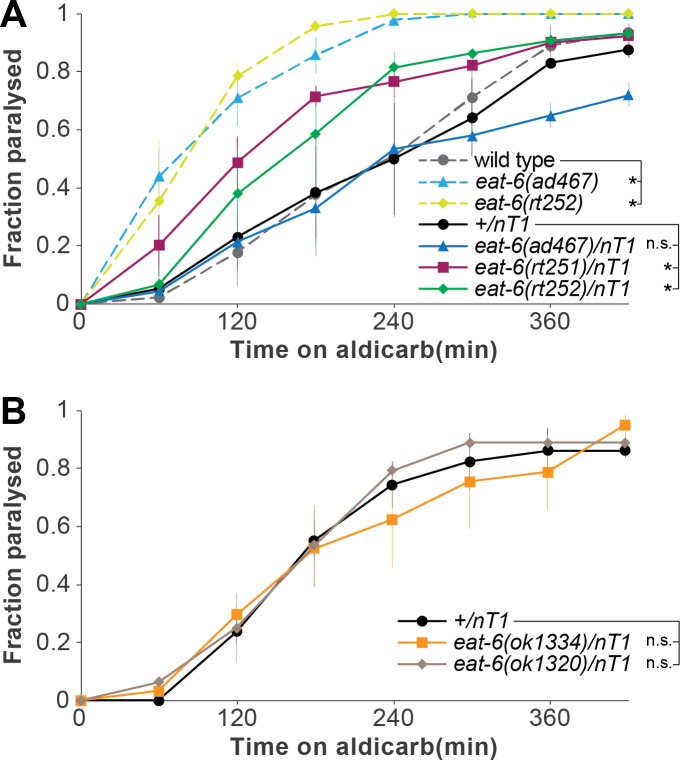
EAT-6 G304S has a dominant negative effect. (A) Animals heterozygous for the G304S mutation were hypersensitive to 1mM aldicarb, whereas the reference *ad467* allele was recessive (3 trials ± SEM, *: log-rank p-value<0.05). (B) Animals with heterozygous *eat-6* complete loss of function alleles did not exhibit neuromuscular defects (2 trials ± SEM; n.s.:not significant, p-value>0.05).

**Fig 3:**
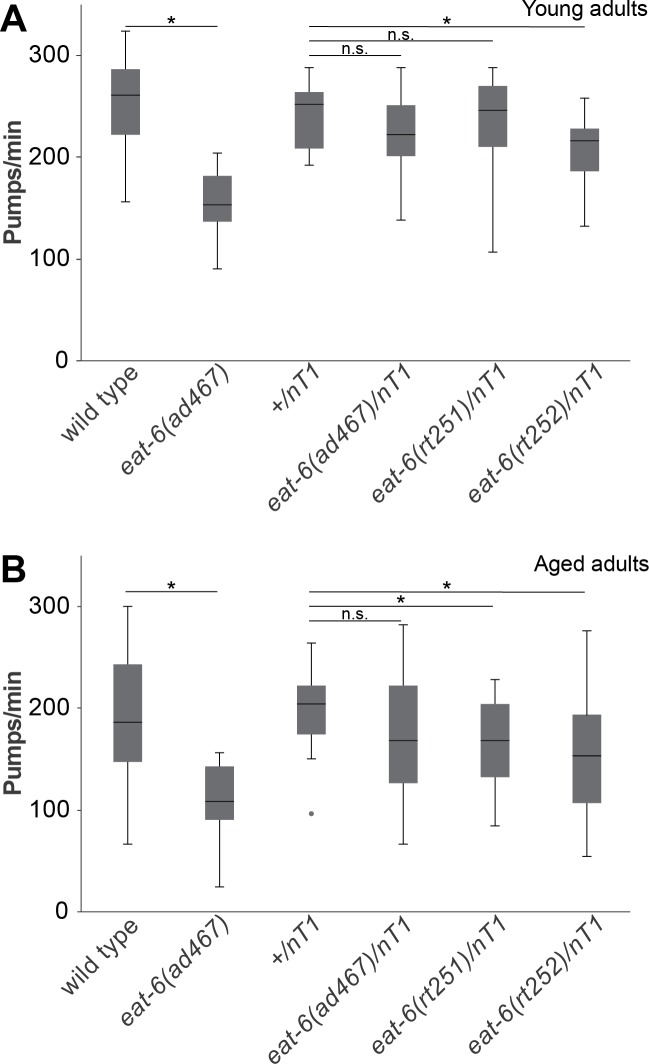
The dominant negative effects of G304S increase over time. (A) *eat-6(rt252)* heterozygous animals had decreased pumping rates as young adults. Pumping defects caused by *ad467* were recessive. However, (B) both *eat-6(rt251)* and *eat-6(rt252)* mutants had reduced pumping rates when aged to 8 days of adulthood (median, flanking quartiles in Tukey format; 3 trials, *: Mann-Whitney U-test (two-tailed) p-value<0.05; n.s.: not significant, p-value>0.05).

The patient is heterozygous for the *de novo* G314S mutation in ATP1A3. If the mutation contributes to symptoms, it would be dominant or haploinsufficient. Based on this, we determined if *C*. *elegans* heterozygous for the G304S mutation (*rt251/nT1* and *rt252/nT1*) would exhibit defects in behavioural paradigms tested here. We confirmed that heterozygotes for the reference *eat-6* partial loss of function allele *ad467* (*ad467/nT1*) were not different from control animals in aldicarb-induced paralysis rates (Fig 2A; p-value for *ad467/nT1* v. +/nT1 = 0.204) and pharyngeal pumping rates (Fig 3A; p-value for *ad467/nT1* v. +/nT1 = 0.219), suggesting that incomplete loss of EAT-6 function does not cause dominant defects. By contrast, animals heterozygous for G304S differed from controls. Both *eat-6(rt251)* and *eat-6(rt252)* heterozygous animals were hypersensitive to aldicarb compared to appropriate controls *(+/nT1)* (Fig 2A; p-value for *rt251/nT1* v. *+/nT1* = 8.327E-9, p-value for *rt252/nT1* v. *+/nT1* = 8.587E-13). This could be due to haploinsufficiency of the G304S mutation. Given that the reference *eat-6* allele *ad467* causes incomplete loss of function, we sought to determine whether complete loss of function alleles of *eat-6* would have a dominant effect. First, we examined animals lacking *eat-6* completely and observed that *eat-6* null alleles cause lethality. Since animals homozygous for the G304S mutation are viable, we conclude that the mutation does not cause a complete loss of function. We then determined that the response of animals heterozygous for these alleles (*ok1320/nT1* and *ok1334/nT1*)did not differ from the wild type response to aldicarb (Fig 2B; p-value for *ok1334/nT1* v. *+/nT1* = 0.981, p-value for *ok1320/nT1* v. *+/nT1* = 0.325). We conclude that *eat-6* complete loss of function do not cause haploinsufficiency.

In pharyngeal pumping, only *eat-6(rt252)* heterozygous animals were significantly different from controls (Fig 3A; p-value for *rt251/nT1* v. *+/nT1* = 0.849, p-value for *rt252/nT1* v. *+/nT1* = 0.002). This suggests that the G304S mutation might cause defects in the presence of a wild type copy of the gene. Since the *eat-6(rt251)* heterozygous animals were not defective in pharyngeal pumping as young adults, we examined whether aging might aggravate the EAT-6 G304S defect. *C*. *elegans* lifespan is roughly 3 weeks under standard laboratory conditions. We determined that 8 day-old animals heterozygous for the G304S mutation had significantly reduced pumping rates compared to age-matched controls (Fig 3B, p-value for *rt251/nT1* v. *+/nT1* = 0.002, p-value for *rt252/nT1* v. *+/nT1* = 0.0002). These results indicate that dominant defects caused by the G304S mutation worsen over time.

## Discussion

Using established behavioural assays and pharmacological manipulations, we demonstrated that the G304S mutation in EAT-6, orthologous to G316S in ATP1A3, likely causes loss of protein function. Animals homozygous for the G304S mutation resemble canonical *eat-6(ad467)* partial loss of function animals: they exhibited decreased pumping rates, and accelerated paralysis when treated with the acetylcholinesterase inhibitor aldicarb. Sweadner *et al*. reported that the activity of the Na^+^/K^+^ ATPase is reduced by the G316S mutation. Na+/K+ ATPases are necessary for cell survival. In HEK293 cells, when the endogenous pump is inhibited by ouabain, exogenous overexpression of the mutant pump is not sufficient for complete viability of the cells. These results show that the mutation causes a loss of function [2]. Behavioral and genetic results presented here are consistent with the G304S mutation in *C*. *elegans eat-6* acting as a hypermorphic or hypomorphic allele. However, examination of results from Sweadner et al. suggests that G316S is a hypomorphic allele resulting in decreased function.

Furthermore, we suggest that the ATP1A3 G316S mutation likely has a dominant negative effect. Unlike *in vitro* overexpression experiments conducted by Sweadner *et al*., we were able to analyse the effect of a single copy of the mutated gene in the presence of a wild type copy. These heterozygous animals exhibited similar defects as homozygous mutants. This effect was not observed in *eat-6* partial or complete loss of function animals, highlighting the significance the G316 residue in protein function.

The two alleles created here were not identical in all assays. They differed significantly in their effect on pharyngeal pumping rates. This may underline the importance of controlling for background mutations. The standard in the *C*. *elegans* field to confirm gene function requires either rescue of phenotypic defects or concordant results with more than one allele. Here, isolation of two independent alleles satisfied this necessity. The differences observed between *eat-6(rt251)* and *eat-6(rt252)* alleles may be due to one or more background mutations that impact pumping rates. However, given the similarity in aldicarb hypersensitivity induced by these two alleles, the alteration of pharyngeal pumping rates, and aggravation with time, we believe that the G304S mutation in *C*. *elegans* EAT-6 is a conditional, dominant loss of function mutation. The phenotypes observed in these mutants might be due to the creation of off-target mutations[21]; however, since the closest matches to our sgRNA binding sequence contain at least four mismatches and given the small size of the *C*. *elegans* genome[18], this is highly unlikely.

Studying the molecular causes of rare diseases facilitates correct diagnosis and helps delineate the function of the proteins involved, which may lead to selection or development of better therapies. Advances in exome sequencing can provide the genetic information necessary to study rare diseases; however, assigning the causality between exonic mutations and these diseases is critical to understanding symptom basis and prognosis.

*C*. *elegans* is a fast and reliable model system for studying neurological diseases given the conservation of neuronal genes and pathways, ease of transgenesis, and well-defined behaviours. Here, we modelled a specific *de novo* mutation in the Na+/K+ ATPase subunit ATP1A3 in *C*. *elegans* and determined if it likely contributes to the symptoms the patient exhibited. Based on the results reported here and in the study by Sweadner *et al*. we conclude that the G316S mutation in ATP1A3 likely causes the patient’s syndrome. In addition to this, as explained by *Sweadner et al*., the UBQLN4 mutation that the patient carries might aggravate the symptoms[2]. Modelling this rare disease provides a strategy for modelling other rare diseases and may contribute to developing treatments for these devastating disorders.

## Materials and Methods

### *C*. *elegans* strains

Strains used in this study are: N2 (Bristol wild type strain), GE24 *pha-1(e2123) III*, DA467 *eat-6(ad467) V*, VC3371 *eat-6(ok1320) V/nT1 [qIs51] (IV;V)*, VC836 *eat-6(ok1334) V/nT1 [qIs51] (IV;V)*, HA2900 *eat-6(rt251) V*, HA2901 *eat-6(rt252) V*, HA2932 *+/nT1 [qIs51] (IV;V)*, HA2933 *eat-6(ad467) V /nT1 [qIs51] (IV;V)*, HA2934 *eat-6(rt251) V /nT1 [qIs51] (IV;V)*, HA2935 *eat-6(rt252) V /nT1 [qIs51] (IV;V)*.

### CRISPR/Cas9 microinjection

*eat-6(rt251)* and *eat-6(rt252)* were generated using procedures detailed by Ward, J.D.[23] with modifications noted here. To create the *eat-6* sgRNA plasmid, we used *PU6*::*klp-12_sgRNA* as the template (Addgene #46170;[17]), and amplified it by PCR using the following primers from Eurofins: F: 5’-GGTCCCTATATAATTGAATTGCAAATCTAAATGTTTGCCGCTGTTG-3’ R: 5’-GCCTTATTTTAACTTGCTATTTCTAGCTCTAAAACATGAGGAAAA-3’. Linear PCR product was circularised using T4 DNA ligase (NEB#M0202). Sanger sequencing was used to verify the plasmid sequence. Plasmids were purified using the Qiagen Plasmid Midi Kit. The template for homologous recombination was the oligonucleotide: 5’-CTTCATCCTCGGATACCATTGGCTTACCGCTGTTGTTTTCCTCATCTCGATCATCGTCGCCAACGTCCCAGAAGGATTGATCGCTACCG-3’

*Microinjection pool*: pJW1285 (50 ng μL^-1^); *pha-1_ssODN200* (50 ng μL^-1^)[23]; *eft-3p*::*Cas9* (50 ng μL^-1^)[17]; PU6::eat*-6*_sgRNA (50 ng μL^-1^); *eat-6* G304S oligonucleotide (50 ng μL^-1^); pCFJ90 (2.5 ng μL^-1^)[24]. The pool was injected into the germline of *pha-1(e2123)* animals reared on nematode growth medium (NGM) seeded with *Escherichia coli* HB101 at the permissive temperature of 15°C. Injected animals were then transferred on NGM plates seeded with *E*. *coli* OP50 and incubated at 25°C. Rescued F_1_ animals were singled onto individual plates and lysed after 2 days of egg laying. Individual F_1_ animals were lysed in 3 μL lysis buffer containing proteinase K and *eat-6* gene was amplified by PCR and sequenced. Both alleles were homozygozed and subsequently used for crosses and behavioural studies. The closest off-target for the sgRNA used in this study -with four mismatches- was *cash-1*, a gene that is not related to Na+/K+ ATPases.

### Behavioural Assays

#### Pharyngeal pumping

Animals were grown at 25°C on NGM plates seeded with *E*. *coli* OP50. Assays in Fig 3B used 8-day old animals (adults); all the others used 3-day old animals (young adults). Assays were conducted at room temperature and carried out blinded as to the genotype. Grinder movement in any axis was scored as a pumping event. The mean pumping rates were calculated from 3 independent trials for each experiment; error bars represent SEM (24≤*n*≤30 for each genotype). Statistical analysis: Mann-Whitney U-Test, two-tailed. The pharyngeal pumping rates for homozygous and heterozygous animals were determined in different sets of experiments and cannot be directly compared.

#### Aldicarb sensitivity

Animals were reared as above. Paralysis caused by 1mM aldicarb (Sigma) was scored as inability to move or pump within 5 seconds after being prodded by a metal wire twice in the tail and then twice in the head region. Mean paralysis rates were calculated from 2 or 3 independent trials; error bars represent SEM (39≤*n*≤47 for each genotype). Statistical analysis: SPSS^TM^ log-rank analysis, pairwise over strata. The response of homozygous *rt252/rt252* animals is significantly different from heterozygous *rt252/nT1* animals. However, we hesitate to draw this conclusion because of the presence of the *nT1* balancer may affect behavior.

## Supporting Information

S1 Fig EAT-6 and ATP1A3 alignment. Alignment for human ATP1A3 (in yellow) and *C*. *elegans* EAT-6 (orange). EAT-6 G304 is highlighted in blue and L359 mutated in the *ad467* allele is highlighted in green.
(TIF)

S1 File Raw data for all experiments. (XLSX)
