# Supplementary figures and images for: In Vivo Modelling of ATP1A3 G316S-Induced Ataxia in C. elegans Using CRISPR/Cas9-Mediated Homologous Recombination Reveals Dominant Loss of Function Defects
Source: PLoS One. 2016 Dec 9;11(12):e0167963. doi: 10.1371/journal.pone.0167963 (PMC5148073; doi:10.1371/journal.pone.0167963)

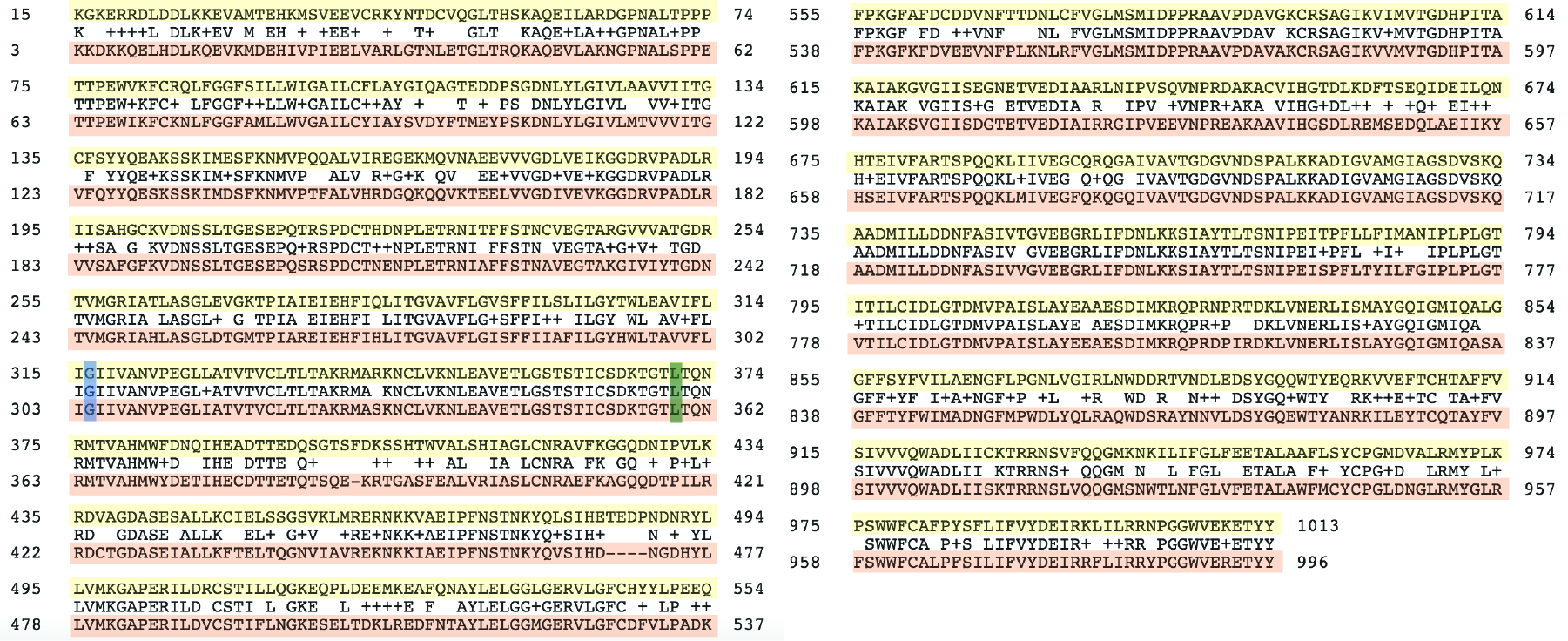

Supplement: S1 Fig — Alignment for human ATP1A3 (in yellow) and C. elegans EAT-6 (orange). EAT-6 G304 is highlighted in blue and L359 mutated in the ad467 allele is highlighted in green. (TIF) [file pone.0167963.s001.tif]
